# Relationships of rapid weight regain variables in professional MMA fighters between 2018–2023

**DOI:** 10.1080/15502783.2025.2550158

**Published:** 2025-08-25

**Authors:** Savannah Calleja, Peter Byers, Casandra Evans, Gabriel J. Sanders, Tobin Silver, Corey A. Peacock, Tony Ricci, Jose Antonio

**Affiliations:** a Nova Southeastern University, Department of Health and Human Performance, Davie, FL, USA; b University of Cincinnati, Exercise Science, Cincinnati, OH, USA

**Keywords:** Weight class, weight regain, height, MMA

## Abstract

**Background:**

Mixed Martial Arts (MMA) athletes from the Ultimate Fighting Championship (UFC) and Bellator undergo rapid weight regain (RWRG) practices between the official weigh-in and fight night. These athletes are categorized into weight classes and participate in similar weight-cutting regimens. While there have been correlational studies regarding anthropometric measurements and physiological profiles, little research has investigated the relationship between anthropometric measurements and RWRG. Therefore, this study examines the relationships of RWRG variables in professional MMA fighters between the official weigh-in and fight night.

**Methods:**

Data from 245 professional MMA fighters (30.2 ± 4.6 years, 175.4 ± 8.4 cm) competing between 2018 and 2023 were utilized for this study. Fighters had 24–36 hours to rehydrate between the official weigh-in and fight night. Weigh-ins were supervised under the California State Athletic Commission (CSAC) using a commission-calibrated scale. Pearson correlation analyses were utilized to examine the relationships between age, anthropometric variables, and percentage of RWRG. Statistical significance was set at *p* ≤ 0.05.

**Results:**

Pearson correlation analyses revealed significant relationships among age, anthropometric variables, and percentage weight change. Height was strongly and positively correlated with both weight (*r* = 0.800, *p* < 0.001) and fight weight (*r* = 0.795, *p* < 0.001). A weak but significant negative correlation was found between height and percentage weight change (*r* = -0.127, *p* = 0.047). Body weight showed a strong positive correlation with fight weight (*r* = 0.976, *p* < 0.001) and a weak negative correlation with percentage weight change (*r* = -0.249, *p* < 0.001). No other significant relationships were observed (*p* ≥ 0.05).

**Conclusion:**

There are significant correlations between anthropometric variables and RWRG in MMA fighters. The results indicate that taller fighters generally weigh more and compete in heavier weight classes. These findings contribute to the existing literature on correlations between RWRG, height, and weight in MMA athletes. Future research could examine whether RWRG practices differ based on these observed relationships.

